# Caregivers’ views on stigmatization and discrimination of people affected by leprosy in Ghana

**DOI:** 10.1371/journal.pntd.0006219

**Published:** 2018-01-29

**Authors:** Emmanuel Asampong, Mavis Dako–Gyeke, Razak Oduro

**Affiliations:** 1 Department of Social and Behavioural Sciences, School of Public Health, University of Ghana, Legon, Accra, Ghana; 2 Department of Social Work, University of Ghana, Legon, Accra, Ghana; 3 Department of Human Ecology, University of Alberta, Alberta, Canada; Swiss Tropical and Public Health Institute, SWITZERLAND

## Abstract

**Background:**

Leprosy is a condition that has long been associated with stigma and discrimination, even when infected persons have been cured. This paper describes stigma and discrimination as viewed by caregivers who are associated with people affected by leprosy in Ghana.

**Methods:**

A qualitative interview with semi-structured interviews were conducted for twenty caregivers.

**Results:**

Findings indicated that caregivers were of the view that people affected by leprosy in Ghana are stigmatized and discriminated against by the larger society thus making their movements and interactions restricted to the Leprosarium. Besides, employments opportunities are unavailable to them thus making them exposed to financial challenges. The livelihood Empowerment Against poverty (LEAP) money given them is not sufficient for their daily upkeep.

**Conclusion:**

People affected by leprosy in Ghana are stigmatized and therefore find it difficult to interact freely with the public. The associated physical deformities with the disease also tend to impede their ability to relate to the general public. The LEAP cash given to people affected by leprosy is helpful however, it could be enhanced to keep pace with prevailing economic conditions in the country.

## Author summary

In Ghana, the social interpretation of leprosy regardless of the language, culture and tradition engenders stigmatization and discrimination that leads to social rejection and exclusion of persons who have been cured of the disease. Often, these persons are cared for by relatives who happen to live with them in a confined place. From the views of these caregivers, this paper identifies areas of stigmatizing and discriminatory tendencies against people affected by leprosy who reside in a Leprosarium in Accra, the capital city. It is expected that persons suffering from such neglected tropical disease would be empowered to enable them go about their daily routines without the fear of being rejected. Besides, the intensification of public education about curing tendencies of stigma becomes paramount.

## Introduction

The understanding and management of leprosy has seen tremendous improvement over the years. This accomplishment can be attributed to the increasing availability of the Multi-Drug Therapy (MDT) that has enabled many sufferers globally to be cured of the disease. Over the years in Ghana, efforts put in place have realized a reduction of 40,000 registered leprosy cases in 1948 [1] to 345 registered cases in 2014. Besides, new cases detected was put at 366 [2] an indication that the disease is still prevalent in Ghana albeit in relatively small numbers. For instance, there is report of the prevalence of leprosy in the Sene Distirct in the Brong Ahafo Region [3], and some other districts in the Volta and Northern Regions [4].

The disease can be cured as long as the precise diagnosis is made and treatment initiated with the appropriate medication [3]. Consequently, infected persons can receive medical attention and be cured in a year and get back to normal life, especially when the disease does not reach the disabling stage [4]. Unfortunately, for many sufferers of leprosy, medical attention is sought when the disease has reached an advanced stage and caused severe deformities and disabilities [5]. For example, persons who get treatment may end up having some physical deformities such as scarring on parts of their body [6]. These physical impairments result in exacerbating the stigma associated with the disease thus sustaining the cycle of stigmatization [4]. In Ghana, the social interpretation of the disease regardless of the language, culture and tradition engenders stigmatization. The Akan language for instance, that is spoken mostly from the south to the north of the country, the condition is commonly called “KWATA” a term that is associated with apprehension for dealing with leprosy-affected people. Again, in the capital city where the Ga language is spoken by the indigenes, it is called “KPITI” which also elicits similar apprehension. This tendency tends to entrench the stigma associated with the disease. Leprosy has therefore become a disease of public health concern because of its association with stigmatization of persons suffering from the disease [7].

The concept of stigma is defined as an attribute that is deeply discrediting within a particular social interaction [8]. Following from that, stigma has been described as “the social devaluation of a person” [9]. As long as individuals who have leprosy are stigmatized, tendencies of discrimination are exhibited towards them. These discriminatory tendencies lead to disadvantages in many areas of life that include personal relationships and work. These individuals tend to accept the situation thus culminating in an acceptance of the discrediting prejudices held against them, which tend to diminish their self-esteem, which also leads to feelings of shame, a sense of alienation and social withdrawal [10, 11].

Over the years, advancements have been made in the treatment of the disease and in Ghana, a number of people who once had the disease have been cured of it. That notwithstanding, stigma is still high for these people who are mostly unable to integrate back into their previous communities for fear of rejection. Some studies have shown that the stigma associated with the disease is heightened by the physical deformities associated with the disease [12]. Clearly, the lack of understanding and knowledge about leprosy increases the misconceptions about the transmission and treatment of the disease [13]. In effect, people affected by leprosy continue to experience negative connotations such that they are perceived to be lepers, a terminology that in itself is synonymous to stigma [4].

In spite of the cured status of persons living at the Weija Leprosarium in Ghana, they continue to experience stigma and discrimination because the social pathology of the disease continues to be associated with them. For example, people affected by leprosy at the Leprosarium reported experiences of stigma and discrimination from their families, friends, healthcare providers and community members [14]. In the lives of people affected by leprosy are caregivers who take on different roles in the daily upkeep of the latter [15]. These caregivers are among those who come into close contact with people affected by leprosy and also interact freely with the general public. Per their caregiving role, they observe at firsthand stigmatizing and discriminatory tendencies that are exhibited by the public towards persons who have been cured of leprosy. It is of paramount significance to be aware of the views of these caregivers so as to help develop an appropriate and comprehensive intervention to address stigma and discrimination tendencies by the public towards persons affected by leprosy. This study therefore sought to understand those experiences from the perspectives of the caregivers at the Weija Leprosarium in Accra, so as to confirm that people affected by leprosy in Ghana do experience stigma and discrimination.

### Theoretical background

This study draws on the Modified Labeling Theory (MLT) developed by Link and colleagues [16, 17], and the conceptualized four dimensional mechanisms of perceived stigma [18]. Per the MLT, stigma is an internal process that inherently involves the negative responses of persons in the environment, which is defined as the “labeling” behaviors of others.

People in the society tend to be apprehensive coming into contact with people affected by leprosy. Besides, there is hesitation whenever people have to relate freely with people affected by leprosy. The theory proposes that labeled individuals will respond behaviorally to anticipate social rejection. In effect, people affected by leprosy in anticipation of how the society will react towards them would rather keep to themselves and mostly remain in the leprosarium. Further, harmful effects may arise from internalized conceptions of anticipated stigma or from the stigma coping response enacted. Labeling thus may negatively affect one’s psychological state.

The four dimensions of perceived stigma include social rejection (e.g., friends, family, colleagues abandoning not wanting to get close to people affected by leprosy), financial insecurity (e.g., feeling financially inadequate), internalized shame (e.g., feelings of embarrassment about deformities of people affected by leprosy), and social isolation (e.g., limiting social contact due to societal behaviours towards people affected by leprosy). People affected by leprosy at the Weija Leprosarium tend to experience all these dimensions. For people who experience internalized stigma, they may suffer poor psychological wellbeing [19, 20].

## Methods

### Ethics statement

The ethical review and approval of the study was sought from the Ethics Committee for the Humanities (ECH) at the University of Ghana. Permission was also sought from the authorities of the Weija Leprosarium in the Greater Accra Region of Ghana. Furthermore written and informed consent forms were signed or thumb-printed by study participants before they participated in the study. The objectives of the study and study procedures were explained to all participants. In addition, anonymity and confidentially was assured to the participants prior to each interview. Participants were made aware that their participation was entirely voluntary and they had the right to refuse to participate or to withdraw from the study at any time if they so desired. It was explained to the participants that their participation in the study would not pose any risks to them and also refusal to participate will not affect any services provided to their respective cured leper. All information received were anonymized and can therefore not be traced to any particular participant.

### Study context

A qualitative research design was employed for this study because it provides complex social processes that capture important aspects of a phenomenon from the perspective of study participants [21]. Semi-structured in-depth-interviews [22] were carried out with purposively selected caregivers at the Weija Leprosarium. This technique was used because we were interested in informants who have the best knowledge or experience concerning the research topic and with the expectation that each participant will provide unique and rich information of value to the study [23].

The selection criterion was caregivers who had at least one year continuous experience in providing care to the person affected by leprosy. Subsequently, appointments were scheduled with caregivers who were willing to participate in the study at their convenience.

The interviews were conducted and recorded in English, and Twi (a local dialect) that was mostly understood and spoken by participants who could not speak English. The interviews were conducted in a secluded environment within the leprosarium so as to avoid interruptions by other people.

### Data collection

The interviews were conducted in person, with two interviewers and a participant. The two interviewers were linguistically competent in both English and Twi (one of the local dialects). These interviewers took turns to moderate all the interviews. The interviews focused on issues such as stigma and discrimination among people affected by leprosy, challenges they encounter in accessing health care and employment opportunities available as well as support systems available to them. In all, twenty in-depth interviews were conducted. When it was realized that no new further information was obtained, saturation was attained and therefore the interviews were ended [24, 25]. All the interviews were audio-recorded and field notes taken by two research assistants who were fluent in both English and Twi.

### Data analysis

The qualitative responses recorded during the In-depth Interviews (IDIs) were translated verbatim and transcribed by two interviewers into English. Further, the notes taken during the interview sessions were expanded. In a situation where there was disagreement between the two interviewers, the transcripts and the original recordings were reviewed until consensus was reached. The transcripts and expanded notes were stored as files and coded manually for textual analysis in accordance with the principles of grounded theory [24]. Coding was specifically done by placing blocks of text into various nodes based on the categories and subcategories. Using the categories, information was compared across the transcripts based on similar and contrasting views of caregivers’ on stigma and discrimination among people affected by leprosy. The themes were illustrated with verbatim quotes and interpreted based on existing literature.

## Results

### Participants’ characteristics

A total of 20 caregivers took part in the study. They were made up of 8 males and 12 females whose ages ranged from 18 to 70 years. The males were made up of a husband, two sons, and five grandchildren. On the other hand, the females were made up of two wives, three daughters and seven grandchildren. Of the 20 participants, only six had no formal education, the rest of them had some education ranging from primary through to post-secondary. Majority of them had some form of employment. Whereas 16 of them indicated that they were Christians, 3 were Muslims and only 1 traditionalist.

### Caregivers’ perceptions about stigma and discrimination

The caregivers demonstrated knowledge of stigma and discrimination through their own behaviours prior to the care giving role and their observations of the general public. As caregivers of people affected by leprosy, participants perceived that they themselves at the initial stage, had strong stigmatizing and discriminatory tendencies about the leprosarium and the leprosy disease.

For many people who come into contact with the facility, their apprehensions are expressed in diverse ways. When the contact is with the residents of the facility, then there is the likelihood of heightened apprehension in spite of the cured status of the residents at the leprosarium. The participants in this study indicated these apprehensions as they noted:

“….the first time I went to Weija (Leprosarium) I did not eat there… It was because I didn’t feel comfortable seeing some of the deformities on the people affected by leprosy. I couldn’t imagine eating at this place, having in mind some of those scary deformities”

Similarly, other participants made the following statements:

“…when I came, it was difficult for me. Now, when she eats and some remains, I am able to eat it. Me it does not do anything to me”.

“Initially people couldn’t even eat, they were scared to eat here but now people come and they eat here”.

The above statements attest to the initial apprehensions that are exhibited by people who come into contact with people affected by leprosy. Over time however, these apprehensions tend to dissipate suggesting that continuous interaction with the leprosarium would get rid of the fear that is otherwise associated with the leprosarium and persons who have been cured of the disease.

Whereas caregivers over time get used to the leprosarium and the inmates, the public on the other hand continue to exhibit stigmatizing and discriminatory behaviours towards people affected by leprosy. These behaviours reinforce the social unacceptability of people affected by leprosy. Caregivers were of the view that the public through their actions make it obvious that people affected by leprosy are stigmatized. Participants expressed sentiments that indicate that people affected by leprosy and anything associated with them tend to be devalued. The following statements made by participants emphasize that notion:

“Yes there is this question mark about it in the sense that people are not forth–coming when they hear of lepers".

“They were even producing soap but it collapsed in the sense that people had the mentality that how can somebody with this disease at the end of the day do soap for you to buy”.

“…I remember one time some big people came to Weija, it’s over a year ago, I said follow me and shake hands with the Lepers and they said “next time, next time”.

It is clear from the statements above that stigma and discrimination exhibited towards people affected by leprosy is extended to products they sold thus curbing their desire to engage in any income generating activities. The fore-going expressions of the caregivers are manifestations of the theoretical dimensions of social rejection, financial insecurity, and social isolation that people affected by leprosy experience in their lives.

### People affected by leprosy access to health-care services

Caregivers noted that people affected by leprosy could access health care services from the clinic within the Leprosarium. This clinic was purposely built with the support and funds from a Philanthropist to attend solely to people affected by leprosy when it had to do with very minor cases. Besides, there is a Municipal Hospital that is adjacent to the Leprosarium where people affected by leprosy are sent when their health condition is beyond the capacity of the clinic. In instances where their conditions require specialized attention, they are sent to major health facilities in the city. Apart from the clinic within the Leprosarium that provided care without any show of stigma, caregivers were of the view that people affected by leprosy experienced stigma and discrimination when they attend to other health care facilities, thus reinforcing the social rejection experienced by people affected by leprosy A caregiver for example in a statement said:

“I took somebody to the hospital and the doctor just refused to take care of him, he didn’t tell me to my face but he left the patient….the next day, he was dead”.

In a similar way, another caregiver also recounted an experience she witnessed while she had taken a person affected by leprosy to a major hospital for a surgical operation.

“Health care professionals in this major hospital were reluctant to get close to the patient… Hmm, it was clear that they did not want touch her because of the sores on her an I just wondered how a human being could relate like that to another human being”.

### Health-care financing

For all the people affected by leprosy who participated in the study, they had been enrolled onto the National Health Insurance Scheme (NHIS) which they found to be helpful. That notwithstanding, there were other additional health costs that NHIS did not cover. In instances like that, people affected by leprosy themselves had to bear the cost. Incidentally, people affected by leprosy were mostly not financially empowered, thereby emphasizing their financial insecurity. As a result, those additional costs had to be borne by the Lepers’ Aid Committee. The following statements by caregivers attest to that arrangement:

“NHIS is good but it doesn’t tackle the detailed issues that it is supposed to…..you know most of these people affected by leprosy are elderly and as you grow, there comes all kinds of health challenges. Some go for eye surgery which is not paid for by NHIS”.

“They are on NHIS but the NHIS does not cover everything like operations so the Lepers Aid Committee through donors and benefactors find monies and pay”.

### People affected by leprosy access to employment

It has always been the right of people to be employed as long as they possess the requisite skills and qualifications needed for a particular job. The expectation therefore is that employment rights of people affected by leprosy be respected. This study revealed that these rights are highly disregarded by most if not all employers. Participants in this study for example mentioned that many employers were unwilling to employ them because of their negative perceptions and beliefs about the disease. When caregivers in this study were asked questions in respect of employment opportunities for people affected by leprosy, their responses suggested that people affected by leprosy were socially rejected in respect of employment that ultimately leads to their financial insecurities. The caregivers made the following statements in their responses:

“…Err no! Who would want to employ a leper”?

“…people wouldn’t accept to be working with a leper even though these ones have been cured of the disease. If you have to talk about the people you work with for example, who wants to say that I am working with a leper”?

As to why people affected by leprosy had difficulty getting employed, caregivers enumerated some concerns that could pass for reasons why employers do not want to engage people affected by leprosy. In the view of the caregivers, employers will do then a lot of good if they were to outline the reasons why they refuse to engage people affected by leprosy. That would have provided some clarity as to who can apply for a job. Participants expressed these sentiments in the following quotes:

I think there should be general information that anybody who has been cured of leprosy cannot apply for so and so job…… that way you know that you cannot be taken because of A, B and C reasons. What I see is that employers will not accept people affected by leprosy but take other people. Why won’t they provide everybody with reasons why people affected by leprosy cannot be employed”?

### Social services available

People who have been cured of leprosy may benefit from social services such as counseling and programmes that seek to empower them in their daily lives. Caregivers interviewed indicated that there were no counseling programmes in place for people affected by leprosy except for the LEAP cash benefits that is given to them periodically. Even that, caregivers were of the view that the amount of money given to people affected by leprosy was inadequate considering the fact that the cost of living lately has increased, more so when people affected by leprosy do not have any other source of income basically because they do not have any job. Caregivers expressed their sentiments in the following statements:

“…they receive 80 pesewas a day for an adult to live on, what you can buy with 80 pesewas a day when the normal wage every day is seven cedis and you expect them to live on 80 pesewas”.

“…because he is married he receives 60 cedis every two months but when he doesn’t receive it in two months then it comes in four months and he takes 120 cedis………just imagine what 120 cedis cannot do much even in one month, let alone four months.”.

It is clear from the foregoing that the only formal support available to the people affected by leprosy is the LEAP programme. In respect of any formal social services instituted for the people affected by leprosy, no such programme exists. This financial support is provided obviously because people affected by leprosy are financially insecured.

### Limitation of study

The results of this study were specifically on caregivers’ perspectives of stigma and discrimination experienced by people affected by leprosy who live in one of the five known leprosaria located in the capital city Accra of Ghana, thus the results may not be generalised to people affected by leprosy in the other leprosaria. Besides, the small numbers of caregivers who participated in the study puts limitation on generalisation of findings to all caregivers of people affected by leprosy in Ghana. It is therefore recommended that future studies will consider caregivers in the other leprosaria in Ghana.

## Discussion

The purpose of this study was to explore caregivers’ perspectives of stigma and discrimination among people affected by leprosy in Ghana. The findings indicate that caregivers are witnesses of stigma and discrimination that people affected by leprosy’ experience. These experiences demonstrate the Modified Labeling Theory [17], that stigma ultimately is an outcome of inherent negative responses in the environment that labels behaviours of others. The four dimensional provision of the theory is a manifestation of experiences that are encountered by people affected by leprosy in Ghana. These are first, the perceived stigma that includes social rejection from relations and the general society. Findings in this study corroborates the long held construction of stigma as a deep rooted phenomenon in societies [26]. Indeed certain entrenched beliefs as well as the lack of knowledge, fear and shame associated with the disease result in the stigmatization of people affected by leprosy [26]. Consequently, irrational behaviours are exhibited towards them. Advances in medicine over the years have turned leprosy into a completely curable disease that can be rendered non-infectious. This is attributed to the introduction and subsequent implementation of the Multi-Drug Therapy (MDT) that has demonstrated to be effective, in curing leprosy [27]. That notwithstanding, many people are unaware of this development thus maintain a certain distance even with people affected by leprosy. The belief therefore continues to be held that one could be infected by getting close to people affected by leprosy. This fear of infection culminates in apprehension, a situation that is similar to a study in Nepal that found the fear of transmission makes people attempt to keep distance with affected persons [28].

Additionally, caregivers in our study identified some hesitation and reluctance on the part of medical practitioners to provide care to people affected by leprosy who are sent to health facilities. These discriminatory tendencies exhibited by health personnel notably some medical Doctors who are assumed to know and understand aspects of the disease including its etiology, causation, means of transmission and curability do not help the course of people affected by leprosy who may require health attention when the need arises. This tendency reinforces stigma associated with the disease [29]. Besides, it is an affront to the World Health Organisation’s (WHO) encouragement for the integration of leprosy into the general health service where leprosy patients should be treated in the same outpatient department as those with any other disease [30]. This can signal to the patients and their communities that leprosy is not a 'different' disease. It has been found that positive attitude of health professionals can contribute significantly in the reduction of stigma due to leprosy [31].

It was evident from our study that people affected by leprosy are financially vulnerable basically because they could not engage in any income generating activities. For those who tried to undertake some economic venture, it could not prevail as soon as patrons realized that persons behind were people affected by leprosy. It is also the case that people affected by leprosy end up having some deformities which in most cases disable them from doing any meaningful work. The consequences of this is loss of earning capacity [32]. To help fill this gap is the LEAP, a social intervention programme instituted in Ghana for vulnerable people. People affected by leprosy have been enrolled unto the programme alright but in the view of caregivers, the amount of money given to them is not sufficient enough. For instance, every cured leper receives and amount of forty eight cedis for two months (equivalent of about fourteen dollars). It is perhaps for this reason that some people affected by leprosy take to begging on the streets as a means to supplement their earnings just as it happens in Ghana.

In the view of caregivers, people affected by leprosy themselves do not express shame in respect of their deformities as long as they remain in the leprosarium where there is some feeling of belonging. This is so because individuals tend to see their personal deformities in other persons as well. It therefore becomes a situation where it is not just one person with some physical deformities. It is rather when they have to interact with the public that they encounter mocking and rude behavior.

Knowing that the deformities that come up due to the disease cannot be reversed, the United Nations General Assembly in the year 2010, unanimously adopted a resolution on the elimination of discrimination against persons affected by leprosy. That resolution was accompanied by principles and guidelines that listed measures to improve the living conditions of such persons. Further, the United Nations Convention on the Rights of Persons with Disabilities (UNCRPD) adopted in the December of 2006 seeks to promote, protect and ensure the full and equal enjoyment of all human rights and fundamental freedoms by all persons with disabilities [33]. People affected by leprosy in Ghana do not seem to enjoy these provisions outlined by the World Body. Their situation is worsened by the secluded and confined environment where they find themselves. About a decade ago for instance, the current location of the leprosarium in Accra appeared remote. In recent times however, population increase and infrastructural developments around the area have brought other communities closer to the leprosarium. In spite of that, there is still the feeling of isolation because walls have been constructed all around the facility, buttressing the phenomenon of social isolation that is still experienced by people affected by leprosy [34].

Following from that, there is limited social contact between people affected by leprosy and the generality of the populace. This social isolation results in some feeling of internal stigma among individual people affected by leprosy. Studies have shown that people who experience internalized stigma tend to suffer poor psychological wellbeing [19, 20]. Psychological well-being connotes lives going well, a combination of feeling good and functioning effectively. In effect, the expected flexibility and creative thinking as well as pro-social behavior and good physical health associated with psychological wellbeing will be lost among people affected by leprosy [35]. Reversing this situation may require an intervention that seeks active participation of people affected by leprosy in programmes that aim at addressing the stigma associated with the disease. This has proven successful in other countries. A Nepalese study for example showed that people affected by leprosy who took part in the Stigma Elimination Programme (STEP) [36] were less stigmatized and participated more in the community than those who did not take part. Besides, STEP participants were more empowered and became change agents in their own communities. This kind of intervention has been proven to be effective, as demonstrated in Ethiopia [37].

### Conclusion

Caregivers interviewed in this study had an understanding of the extent of stigma and discrimination experienced by people affected by leprosy in the Weija Leprosarium. These experiences were generally in the areas of social rejection whereby people see people affected by leprosy as outcasts and therefore, would not want to be close with them. The manifestation of this rejection is the area of social isolation that people affected by leprosy find themselves. The result of this is the limited or no interaction that take place between them and the society in general. People affected by leprosy also had physical deformities which they are not comfortable with especially when they come into contact with the public. In addition, people affected by leprosy receive some amount of money periodically. In their estimation, those amounts could be enhanced to enable them live meaningfully.

### Implications of the study

Knowing that caregivers play an important role especially for persons with stigmatized conditions, this study brings to the fore the need for concerted efforts to reduce stigma and discrimination at the community level with subsequent extensions to generality of the population. This would mean consciously involving caregivers of people affected by leprosy in such activities where they can share their experiences as in getting close to people affected by leprosy will not make one to be infected with the disease. This will help disabuse the minds of people in respect of the negative perceptions about leprosy. There is equally the need for psychosocial interventions to be adopted for people affected by leprosy. This should include activities that seek to empower them to get over feelings of alienation, and their ability to deal with negative reactions from society. Finally, the social benefits in the form of the LEAP that is provided to people affected by leprosy can be leveraged in line with the minimum wage that prevails in Ghana. That way, whenever increments are announced, people affected by leprosy can also be assured that they will get an amount of money that could improve their barest standard of living.
